# Behavioral and neural evidence for perceptual predictions in social interactions

**DOI:** 10.1162/IMAG.a.38

**Published:** 2025-06-11

**Authors:** Juanzhi Lu, Lars Riecke, Beatrice de Gelder

**Affiliations:** Department of Cognitive Neuroscience, Faculty of Psychology and Neuroscience, Maastricht University, Maastricht, Limburg, The Netherlands

**Keywords:** social interaction, prediction, body expression, action prediction, N170, N300

## Abstract

The ability to predict others’ behavior is crucial for social interactions. The goal of the present study was to test whether predictions are derived during observation of social interactions and how these predictions influence the whole-body emotional expressions of the agents are perceived. Using a novel paradigm, we induced social predictions in participants by presenting them with a short video of a social interaction in which a person approached another person and greeted him by touching the shoulder in either a neutral or an aggressive fashion. The video was followed by a still image showing a later stage in the interaction and we measured participants’ behavioral and neural responses to the still image, which was either congruent or incongruent with the emotional valence of the touching. We varied the strength of the induced predictions by parametrically reducing the saliency of emotional cues in the video. Behaviorally, we found that reducing the emotional cues in the video led to a significant decrease in participants’ ability to correctly judge the appropriateness of the emotional reaction in the image. At the neural level, EEG recordings revealed that observing an angry reaction elicited significantly larger N170 amplitudes than observing a neutral reaction. This emotion effect was only found in the high prediction condition (where the context in the preceding video was intact and clear), not in the mid and low prediction conditions. We further found that incongruent conditions elicited larger N300 amplitudes than congruent conditions only for the neutral images. Our findings provide evidence that viewing the initial stages of social interactions triggers predictions about their outcome in early cortical processing stages.

## 1 Introduction

Primates live in complex social networks that are built and maintained by interactions between the members. The primate brain is fine-tuned to perceive nonverbal communication signals from conspecifics. In the domain of vision, social signals are predominantly provided by movements of the face and the body, whether these are displayed by single agents or in interactions. The pioneering research by Heider and Simmel (Heider & Simmel, 1944) demonstrated that humans discern intricate details about others’ interactions based on simple movement cues. In the last two decades, cognitive and affective neuroscientists have started exploring the brain basis of the competences required to engage actively in social interactions and to understand the meaning of observed social interactions (Poyo Solanas & de Gelder, 2025). The centrality of social interaction is underscored by findings showing that an individual’s expressive postures are judged differently depending on whether they are viewed as part of an interaction with another individual. Using well-controlled computer animations,Christensen et al. (2024)showed that the emotional expression of an individual agent is perceived differently when the agent is shown in isolation versus as part of a social interaction. Another behavioral study found that emotions were perceived differently in a social interaction context in which two agents interacted versus did not interact (Abramson et al., 2021). Participants were instructed to categorize the target agent’s emotions (either fear or anger), with the other agent serving as contextual cues. It was found that recognizing fear was easier when participants interacted with an angry emotion compared to a fearful emotion. This effect was observed when participants viewed body or body-face compound stimuli, but not when they viewed faces alone. These studies indicate that body gestures and movements play an important role in emotion perception during social interaction.

Research on the neural basis of affective signals from whole-body postures and movements is still a relatively underexplored field (de Gelder, 2006;de Gelder & Solanas, 2021). Functional magnetic resonance imaging (fMRI) and electroencephalography (EEG) studies have shown that the brain is fine-tuned to details of whole-body postures and movements. Furthermore, observers are not passively registering the visual input from whole-body expressions, but the brain is actively preparing for an adaptive response, such as when a defensive reaction is called for (de Gelder et al., 2004). Importantly, for many familiar actions, once the goals of the action are understood, the end stages can be successfully predicted, as shown in studies comparing basketball novices versus experts. The latter needed less information to accurately predict where a ball was going to land (Abreu et al., 2012; Özkan et al.,^2019^). This ability to predict the outcome of an ongoing action is especially relevant when we observe two agents in the course of a social interaction (McMahon & Isik, 2023). A study byEpperlein et al. (2022)used video clips divided into two parts. Only the first part was shown to participants, depicting real-life interactions between dyads. The clip was interrupted 10 frames before a social interaction took place, and participants were asked to predict the outcome of the observed interaction. The study found that participants were less accurate in predicting outcomes in an aggressive context compared to a playful or neutral context, suggesting that predictions depend on the emotional information available during social interactions. In the present study, we used a similar paradigm, presenting only the first part of the social interaction video to elicit social predictions in participants. However, unlike Epperlein et al.’s study, we also presented the outcome of the social interaction after the short video to examine how social prediction influences the processing of subsequent social information.

A few studies have examined how prediction operates during neural processing of emotional stimuli and found effects on various ERP components (Baker et al., 2023;Vogel et al., 2015). The N170 is an early ERP component that occurs around 180 ms in the temporal regions. Previous studies have found that it is involved in the encoding of not only face stimuli but also body stimuli (Baker et al., 2023;Calbi et al., 2017;He et al., 2018;Stekelenburg & de Gelder, 2004;Van Heijnsbergen et al., 2007). Some studies have found effects of emotional expression on the body-evoked N170 (Lu et al., 2023), while others have not (Stekelenburg & de Gelder, 2004;Van Heijnsbergen et al., 2007). The N300 is a mid-late ERP component that peaks around 300 ms in the frontal regions following the onset of visual stimuli (Kumar et al., 2021).Baker et al. (2023)investigated N170 and N300 responses to face stimuli and found that they are sensitive to emotion-prediction errors, showing stronger responses to unpredictable facial emotional expressions than predictable ones. Similarly,Vogel et al. (2015)found that the mismatch negativity (MMN), a mid-latency event-related potential (ERP) component thought to reflect regularity violations, is sensitive to prediction errors based on facial emotional expressions. Their study showed that incongruent emotional faces (e.g., a neutral face followed by a fearful face) elicited larger MMN amplitudes compared to congruent faces (e.g., a neutral face followed by another neutral face).

A related study investigated the N400, a negative-going component that peaks around 400 ms and reflects violation detection, emotional incongruence, and prediction error (Balconi & Caldiroli, 2011;Hodapp & Rabovsky, 2021;Yu et al., 2022). It was found that perceiving two consecutive emotional expressions elicits a stronger N400 response when the two expressions are incongruent rather than congruent (Calbi et al., 2017). This effect was observed regardless of whether the expression was conveyed by still images of the face or the body, and it might hint at a prediction error response.

Taken together, the N300 and N400 may serve as neural markers of violations of higher-order visual predictions, whereas the N170 may specifically reflect the visual processing of bodies. Given the previous observation of an emotion-prediction effect on the face-evoked N170 (Baker et al., 2023), it remains an open question whether the body-evoked N170 is influenced by emotion predictions during the early stages of observing social interactions. Additionally, it is still unclear how the prediction error effect occurs in the mid-to-late stage when processing dyadic body interactions.

We hypothesized that: 1) Observers of a social interaction derive predictions from their observations about the outcome of the interaction; and 2) These putative social predictions automatically and rapidly influence how the outcome of the ongoing social interaction is perceived. We tested our hypotheses with a novel paradigm: Participants watched a short video clip of a social interaction between two agents, in which agent A approached agent B and touched him on the shoulder, whereupon agent B turned around to face agent A. The videos were stopped before the end and then followed by a still probe image, which was the final frame of the full clip disclosing agent B’s reaction to the interaction. A still image rather than dynamic stimulus was used for the probe to obtain phase-locked responses that would give rise to a clear ERP. In the perceptual task, participants judged the appropriateness of the agents’ reaction from the agent’s bodily expression. For the neural measures, we focused on the ERP components N170, N300, and N400, as reviewed above. By presenting the video clip prior to the still probe we could temporally separate the putative prediction effects of the video from its (shorter-lived) sensory effects. To investigate the impact of social prediction on observing social interactions, we varied both the strength and the correctness of the predictions that observers could derive from the clip. Prediction strength was varied across three levels as follows: in the main “high prediction” condition, the video clearly showed how agent A approached and touched agent B. In the “mid prediction” condition, social interaction information was reduced by backward presentation of the video. Finally, in the “low prediction” condition, each video frame was scrambled, effectively removing any social cues from the video and preventing emotion prediction. These video manipulations were chosen based on prior informal observations suggesting that the different video edits (time-reversal and scrambling) gradually reduce how accurately the adequacy of agent A and B’s action and reaction can be perceived.

Prediction correctness, referred to below as prediction error, was varied by manipulating the emotional congruence between the probe image and the preceding video. This was implemented by preceding each probe condition (image of a neutral or an angry reaction; see above) with either a “neutral” video (in which agent A gently touched agent B’s shoulder) or an “angry” video (in which agent A abruptly pulled agent B’s shoulder). The incongruent condition was designed to trigger prediction errors in participants.

We expected that: 1) If observers of a social interaction derive predictions from it about its outcome, our participants should show more accurate responses in the perceptual task when the preceding clip allows for stronger predictions. 2) If these social predictions influence the processing of the ongoing social interaction, our participants should show neural changes in response to the probe. Specifically, body-related responses (N170) and prediction-related responses (N300 and N400) should reflect variations in prediction strength and prediction errors.

## 2 Methods

### 2.1 Participants

Thirty healthy participants were recruited from the student population at Maastricht University. Two participants’ data were rejected because one participant did not follow the task instructions and another participant’s ERPs data (N170, N300 and N400) exceeded 3 standard deviations (SD) above the mean. Twenty-eight participants’ data were included in the analysis (aged 19–34 years, 24.0 ± 4.9 (mean ± SD); 14 male and 14 female; one left-handed). All participants had normal or corrected-to-normal vision, and no history of brain injury, psychiatric disorders, or current use of psychotropic medication. Before the experiment, participants provided written consent. They received compensation of 7.5 Euros or one study credit point for their participation. The Ethics Committee of Maastricht University approved the study, and all procedures adhered to the principles outlined in the Declaration of Helsinki (approval number: OZL_263_16_02_2023).

### 2.2 Stimuli

The stimuli consisted of video clips of social interactions and still images extracted from the end section of the videos. The videos showed a person on the right (agent A) approaching a person on the left (agent B). At the onset, agent B had his/her back turned away from agent A. Agent A approached and touched agent B on the shoulder whereupon agent B reacted to this by turning around toward agent A.

The video recordings were made with ten actors (six females and four males) who were combined to create five gender-matched pairs. For each actor pair, five “angry” social interactions and five “neutral” social interactions were recorded, resulting in ten videos per pair (50 videos in total). We used similar stimuli and postures asChristensen et al. (2024), who also compared angry versus neutral videos. The still images were created by taking the last frame of the video. These images served as the probes for the participants’ task, which was to rate whether the reaction of agent B (to the touch by agent A) was appropriate. The images and videos were processed using Adobe Premiere Pro and all faces were blurred to exclude the influence of facial cues when observing the body interactions. Videos and still images were presented on a black background (size: 1150 × 1088 pixels), covering approximately 15 × 13 degrees of the participants’ visual angle in the experiment. To ensure that participants focused on the interaction between the two actors, they were instructed to fixate a white fixation cross placed at the center of the screen, located between the two actors. The videos are available in theSupplementary Materials.

### 2.3 Experimental design and procedure

**Fig. 1.**
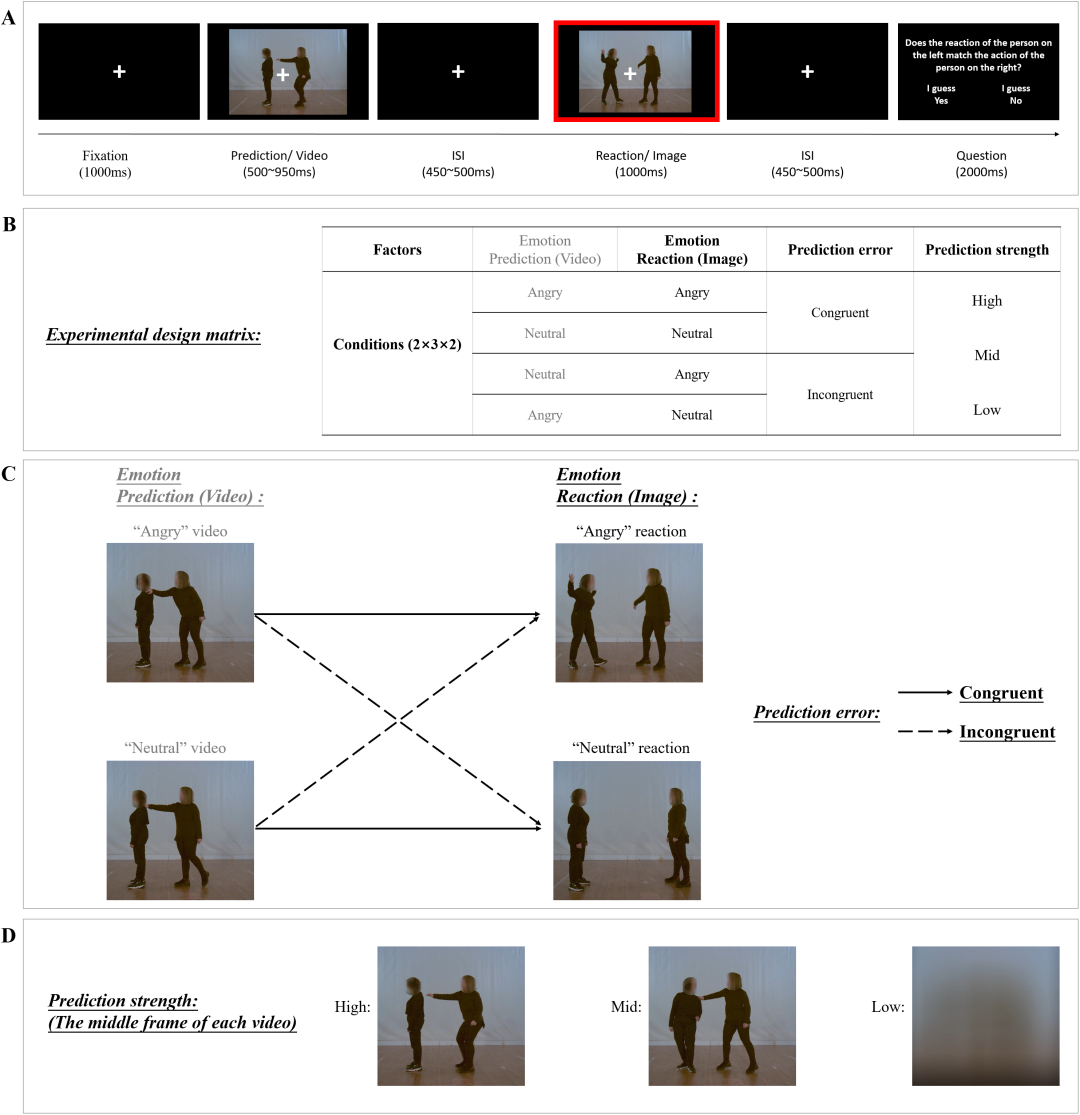
(A-D) Experimental design. (A) Trial procedure. Participants watched a social interaction video followed by a still probe image. At the end of each trial, participants responded to the question on the screen by pressing one of two buttons (yes/no). ERP analysis was time-locked to the still image, see red rectangle. (B) Experimental design matrix. The study used a 2 × 3 × 2 within-subject design with factors emotion reaction (angry, neutral), prediction strength (high, mid, low), and prediction error (congruent, incongruent). (C) Examples of emotional reactions that are included in the matrix of prediction error. The left column of figures shows the middle frame of the “angry” video and the “neutral” video. The right column of figures shows the emotional reaction: the “angry” reaction and the “neutral” reaction. The solid arrows indicate congruent conditions: an angry reaction preceded by an angry video or a neutral reaction preceded by a neutral video. The dashed arrows indicate incongruent conditions: an angry reaction preceded by a neutral video or a neutral reaction preceded by an angry video. (D) Examples of prediction strength in the video, showing the first frame of high, mid, and low conditions.

Each trial started with a 1000-ms fixation period, followed by the presentation of the video. After a short gap (400–500 ms) during which the screen was blank, the probe image was presented for 1000 ms revealing agent B’s reaction. Subsequently, participants were instructed to answer the following question, which was shown on the screen: “Does the reaction of the person on the left match the action of the person on the right?”. Participants chose one of two response alternatives (“I guess yes” and “I guess no”) during this response interval, which lasted 2000 ms (Fig. 1A).

An example of the probe image in the two emotion reaction conditions (angry reaction or neutral reaction) is shown inFigure 1C. The different prediction strength conditions are illustrated inFigure 1D. This manipulation was implemented by playing the video either normally (high prediction), or as time-reversed or scrambled versions. In the backward videos (mid prediction), the visibility of the actors’ movements was preserved, while the interpretation of the social action was hampered. In other words, the clip began with agent A already touching agent B’s shoulder, then releasing the hand, and finally walking away backward (from left to right). In the scrambled videos (low prediction), each frame was masked with Gaussian filters using the Matlab function*imgaussfilt*(filter size: 501, sigma standard deviation: 200) so that both movement and social action information were largely reduced (Fig. 1D).

The manipulation of prediction error was implemented by pairing each video clip with either its original last frame (congruent condition: angry video followed by angry image, or neutral video followed by neutral image) or the last frame of the clip in which the same actors exhibited the other emotion (incongruent condition: angry video followed by neutral image, or neutral video followed by angry image). Example frames from the neutral and angry videos are shown inFigure 1C. Participants’ “Yes” responses on congruent trials and “No” responses on incongruent trials were considered as correct, whereas “No” responses on congruent trials and “Yes” responses on incongruent trials were considered as incorrect.

The study used a fully balanced 2 × 3 × 2 within-subject design. As described above, the first factor was emotion reaction (angry or neutral), the second factor was prediction strength (high, mid, or low), and the third factor was prediction error (emotional valence of image and video: congruent or incongruent). Each of the twelve conditions was presented in 25 unique trials, resulting in a total of 300 trials that were randomly presented in 4 runs, each lasting 7 minutes. Participants took a short break after the first two runs. Before the experiment, participants practiced the task on 24 trials. The whole experiment lasted around 28–35 minutes.

### 2.4 EEG acquisition

EEG data were recorded using an elastic cap with 64 electrodes placed according to the international 10–20 system and sampled at a rate of 1000 Hz (BrainVison Products, Munich, Germany). Electrode Cz was used as the reference during recording, and the forehead electrode (Fp1) was used as a ground electrode. Four electrodes were used to measure the electrooculogram (EOG). Two of them were used as vertical electrooculograms (VEOG). One was placed above the right eye, and another was placed below the right eye. The other two electrodes were used as a horizontal electrooculogram (HEOG), with one placed at the outer canthus of the left eye, and the other at the outer canthus of the right eye. The remaining 60 electrodes included FPz, AFz, Fz, FCz, CPz, Pz, POz, Oz, AF7, AF8, AF3, AF4, F7, F8, F5, F6, F3, F4, F1, F2, FC5, FC6, FC3, FC4, FC1, FC2, T7, T8, C5, C6, C3, C4, C1, C2, TP9, TP10, TP7, TP8, TP9, TP10, CP5, CP6, CP3, CP4, CP1, CP2, P7, P8, P5, P6, P3, P4, P1, P2, PO7, PO8, PO3, PO4, O1, and O2. Impedances for reference and ground were maintained below 5 kOhm and for all other electrodes below 10 kOhm.

### 2.5 EEG data preprocessing

EEG data were preprocessed and analyzed using FieldTrip version 20220104 (Oostenveld et al., 2011) in Matlab R2021b (MathWorks, U.S.). Recordings were first segmented into epochs from 500 ms pre-stimulus (i.e., before the onset of the probe image) to 1500 ms post-stimulus and then filtered with a 0.3–30 Hz band-pass filter. EEG data at each electrode were re-referenced to the average of all electrodes. Artifact rejection was done using independent component analysis (logistic infomax ICA algorithm (Bell & Sejnowski, 1995); on average, 2.97 ± 1.08 (mean ± SD) components were visually identified as artifacts and removed per participant). Moreover, single epochs during which the EEG peak amplitude exceeded 3 SD above/below the mean amplitude were rejected. On average, 71.04% ± 9.14% of trials were preserved and statistically analyzed per participant.

### 2.6 Event-related potential analyses

The EEG analysis focused on neural responses to the probe (reaction) image. Based on previous ERP literature (Chen et al., 2022;Hietanen et al., 2014), we spatially separated the EEG electrodes into a temporal cluster (P7, P8, TP7, TP8, TP9, TP10) and central cluster (FCz, FC1, FC2, Cz, C1, C2, CPz, CP1, CP2), and averaged the channels within each cluster. For each cluster, we pooled all conditions and visually inspected the overall waveform to identify the ERP components of interest (N170, N300, and N400). We observed the strongest N170 in the temporal cluster, and the strongest N300 and N400 in the central cluster. For each of these ERP components, we further visually defined a time window spanning the interval of the ERP, centered on its peak. The resulting time windows were 180–230 ms (N170), 250–350 ms (N300), and 350–500 ms (N400), in line with the aforementioned ERP studies. The mean ERP amplitude was computed as the average response of the cluster within the time window. Baseline correction was applied and involved subtracting the average amplitude in the baseline interval (-200 to 0 ms) from the overall epoch. Trials were averaged for each experimental condition, resulting in ERPs used for further statistical analyses, which were performed using IBM SPSS Statistics 27 (IBM Corp., Armonk, NY, USA).

### 2.7 Statistical analyses

A repeated-measures 2× 3× 2 ANOVA (Emotion reaction: angry/neutral; Prediction strength: high/mid/low; Prediction error: congruent/incongruent) was applied to the behavioral accuracy and the mean ERP amplitudes. Degrees of freedom for F-ratios were corrected with the Greenhouse–Geisser method. Bonferroni’s method was used to correct for multiple comparisons. Statistical results were considered as significant given a corrected*p*-value < 0.05.

## 3 Results

### 3.1 Behavior

The goal of the behavioral analysis was to validate our behavioral paradigm, that is, to verify whether the manipulation of the video induced variations in participants’ predictions. To this end, we put focus on the effects of prediction strength (high, mid and low) and prediction error (congruent and incongruent) on response accuracy (proportion of correct responses), pooled across emotion reaction.

**Fig. 2.**
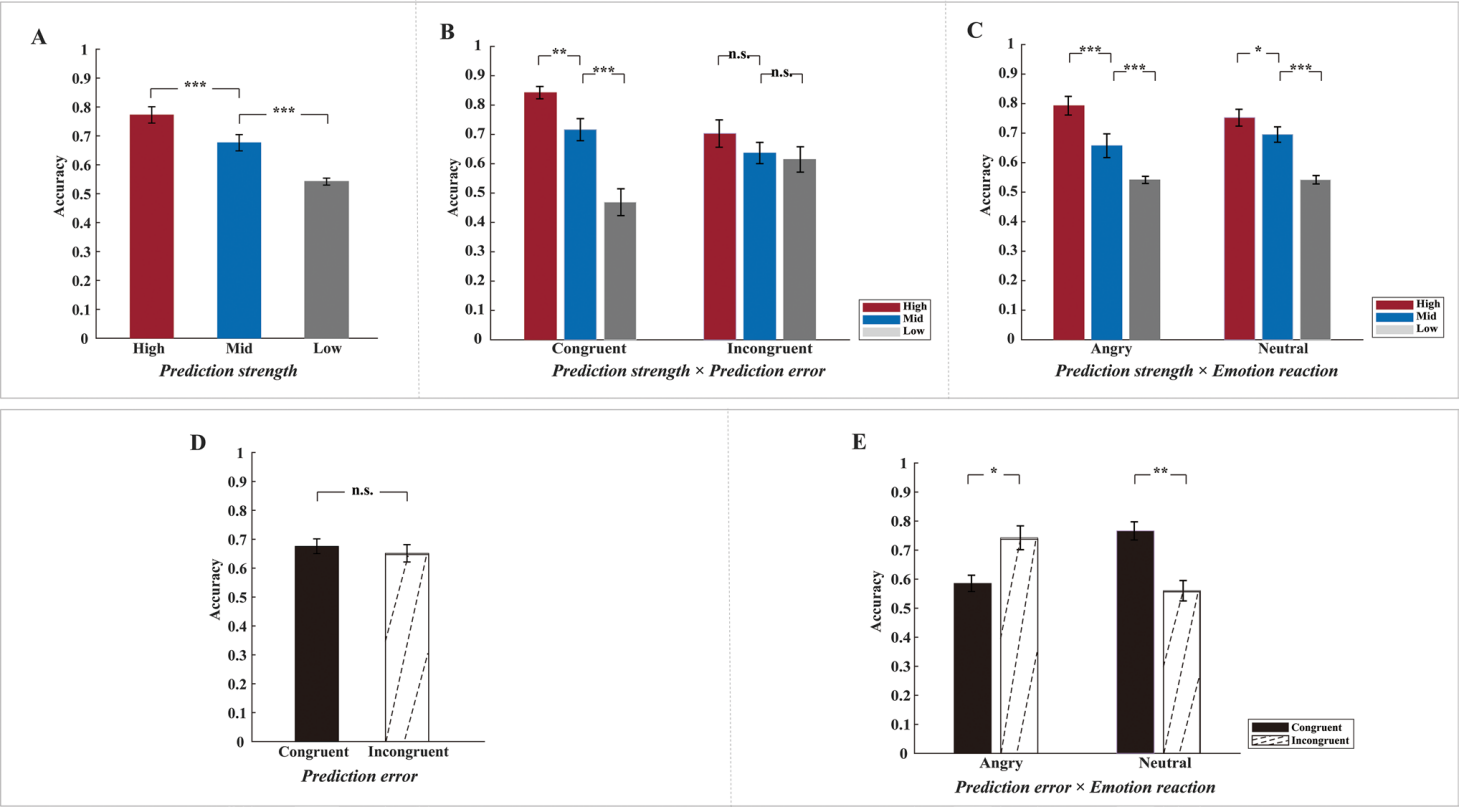
(A) Mean and standard error (SE) across participants of accuracy per prediction strength condition (high, mid and low). (B) Mean and SE of accuracy per prediction strength × prediction error condition. (C) Mean and SE of accuracy per prediction strength × emotion reaction condition. (D) Mean and SE of accuracy per prediction error condition (congruent and incongruent). (E) Mean and SE of accuracy per prediction error × emotion reaction condition. ***p<0.001, **p<0.01, *p<0.05, n.s.: non-significant.

As can be seen fromTable 1, statistical analysis yielded a significant three-way interaction (emotion reaction × prediction strength × prediction error) and two significant two-way interactions (prediction strength × prediction error, emotion reaction × prediction error). Visual inspection revealed that effects of prediction strength had the same direction in all prediction-error conditions (Fig. 2B) and in all emotion-reaction conditions (Fig. 2C); therefore, we further tested for a main effect of prediction strength, which yielded a significant result (*F*(2, 54) = 49.23,*p*< 0.001,*ηp^2^*= 0.65) (high vs. mid:*t*(27) = 6.91,*p*< 0.001; high vs. low:*t*(27) = 8.89,*p*< 0.001; mid vs. low:*t*(27) = 4.85,*p*< 0.001). As expected, accuracy was highest for the high prediction condition (0.77 ± 0.15), followed by the mid-prediction condition (0.68 ± 0.15), and lowest for the low prediction condition (0.54 ± 0.06), seeFigure 2A. These findings indicate that our manipulation of contextual information was effective: reducing the amount of information in the preceding video led to a decrease in prediction accuracy.

**Table 1.** Statistical results: effects on behavior.

Behavioral effects		*F*	*p*	* ηp ^2^*
Three-way interaction	Emotion reaction × prediction Strength × prediction error	9.76	**0.002**	0.27
Two-way interaction	Prediction strength × emotion reaction	2.29	0.122	0.08
	Emotion reaction × prediction error	23.08	**<0.001**	0.46
	Prediction strength × prediction error	6.01	**0.011**	0.18
Main effect	Emotion reaction	0.002	0.962	0.000
	Prediction strength	49.23	**<0.001**	0.65
	Prediction error	0.39	0.536	0.01

Bold values indicate significant effects.

The main effect of prediction error was not significant (*F*(1, 27) = 0.39,*p*= 0.536,*ηp^2^*= 0.01), suggesting that on average, task difficulty did not differ significantly between congruent (0.68 ± 0.14) and incongruent (0.65 ± 0.16) conditions (Fig. 2D). This null result emerged from the aforementioned emotion reaction × prediction error interaction, that is, opposing prediction-error effects in the emotion-reaction conditions (Fig. 2E).

To test whether participants’ choices/accuracy were above chance level, we conducted a one-sample t-test comparing participants’ accuracy in each prediction strength (high/mid/low) and prediction error (congruent/incongruent) condition versus 0.5. We found that the accuracy in all conditions was significantly above chance level (*ps*< 0.002).

### 3.2 ERPs

Our hypothesis concerned the effect of emotional valence (emotion reaction) and its modulation by contextual factors (prediction strength and prediction error). First, we assessed the three-way interaction (emotion reaction × prediction strength × prediction error) and found there was no significant effect for any ERP component. Next, we analyzed the two-way interactions, which revealed a significant emotion reaction × prediction strength interaction for N170, but not the other ERP components, and a significant emotion reaction × prediction error interaction for N300, but not the other ERP components. However, we found no significant prediction strength × prediction error interaction for any ERP component (seeTable 2). In the following sections, we investigated the nature of the observed interactions by testing for simple effects of the interacting factors. We also explored main effects of the factors that showed no significant interactions; these effects were not a focus of the current study and therefore the results are presented in theSupplementary Materials.

**Table 2.** Statistical results: effects on each ERP component.

ERPs effects		N170	N300	N400
Three-way interaction (Emotion reaction × prediction strength × prediction error)	*F*	2.44	0.56	0.83
	*p*	0.097	0.573	0.443
	* ηp ^2^*	0.08	0.02	0.03
Two-way interaction (Prediction strength × emotion reaction)	*F*	3.48	0.92	0.18
	*p*	**0.040**	0.400	0.830
	* ηp ^2^*	0.11	0.03	0.01
Two-way interaction (Emotion reaction × prediction error)	*F*	0.05	6.47	0.11
	*p*	0.829	**0.017**	0.745
	* ηp ^2^*	0.00	0.19	0.00
Two-way interaction (Prediction strength × prediction error)	*F*	2.05	1.32	0.83
	*p*	0.146	0.280	0.040
	* ηp ^2^*	0.07	0.05	0.03

Bold values indicate significant effects of the two-way interaction.

#### 3.2.1 Interaction effect of emotion reaction × prediction strength on N170

To disentangle the observed interaction effect of emotion reaction × prediction strength on N170, we analyzed simple effects of emotion reaction (i.e., per prediction strength), which revealed a significant simple effect of emotion reaction for the high prediction condition (*t*(27) = -5.18,*p*< 0.001) as expected, but not for the mid or low prediction conditions (mid:*t*(27) = -1.41,*p*= 0.507; low:*t*(27) = -1.74,*p*= 0.277). More specifically, angry reactions (-1.45 ± 2.00 µV) elicited larger N170 amplitudes than neutral reactions (-0.52 ± 1.87 µV) in line with previous results (Lu et al., 2023), and interestingly, this enhancing effect occurred only when the images were preceded by a fully intact video (high prediction condition).

**Fig. 3:**
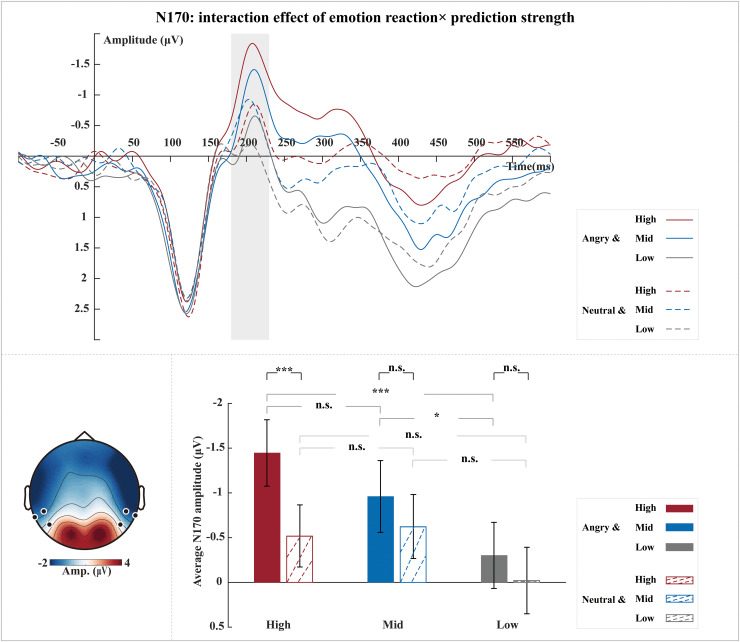
Interaction effect of emotion reaction × prediction strength on N170. Grand-averaged ERP waveforms of N170 per condition (angry-high, neutral-high, angry-mid, neutral-mid, angry-low, and neutral-low) (top). Waveforms were calculated by averaging the data at the electrodes P7, P8, TP7, TP8, TP9, and TP10 (see black dots in scalp map). The shaded rectangle visualizes the time window from which the average ERP amplitude was extracted (180–230 ms). The topographic map was calculated by averaging the data of all conditions within a time window of 180–230 ms after the onset of the probe image (bottom left). Bar plots (bottom right) illustrate the mean and SE across participants of the average N170 amplitude per condition. ***p<0.001, *p<0.05, n.s.: non-significant.

We further observed a significant simple effect of prediction strength for the angry reaction. Both high and mid-prediction were followed by larger N170 amplitudes than low prediction when the following reaction in the probe image was angry; the difference between high and mid-prediction was not significant (Angry reaction: high vs. low:*t*(27) = -4.51,*p*< 0.001; mid vs. lows:*t*(27) = -2.62,*p*= 0.014; high vs. mid:*t*(27) = -2.20,*p*= 0.109). Interestingly, this simple effect of prediction strength was found only for the angry reaction, not for the neutral reaction (Neutral reaction: high vs. low:*t*(27) = -1.76,*p*= 0.272; mid vs. low:*t*(27) = -1.88,*p*= 0.214; high vs. mid:*t*(27) = 0.59,*p*= 1.000) (Fig. 3).

#### 3.2.2 Interaction effect of emotion reaction and prediction error on N300

**Fig. 4.**
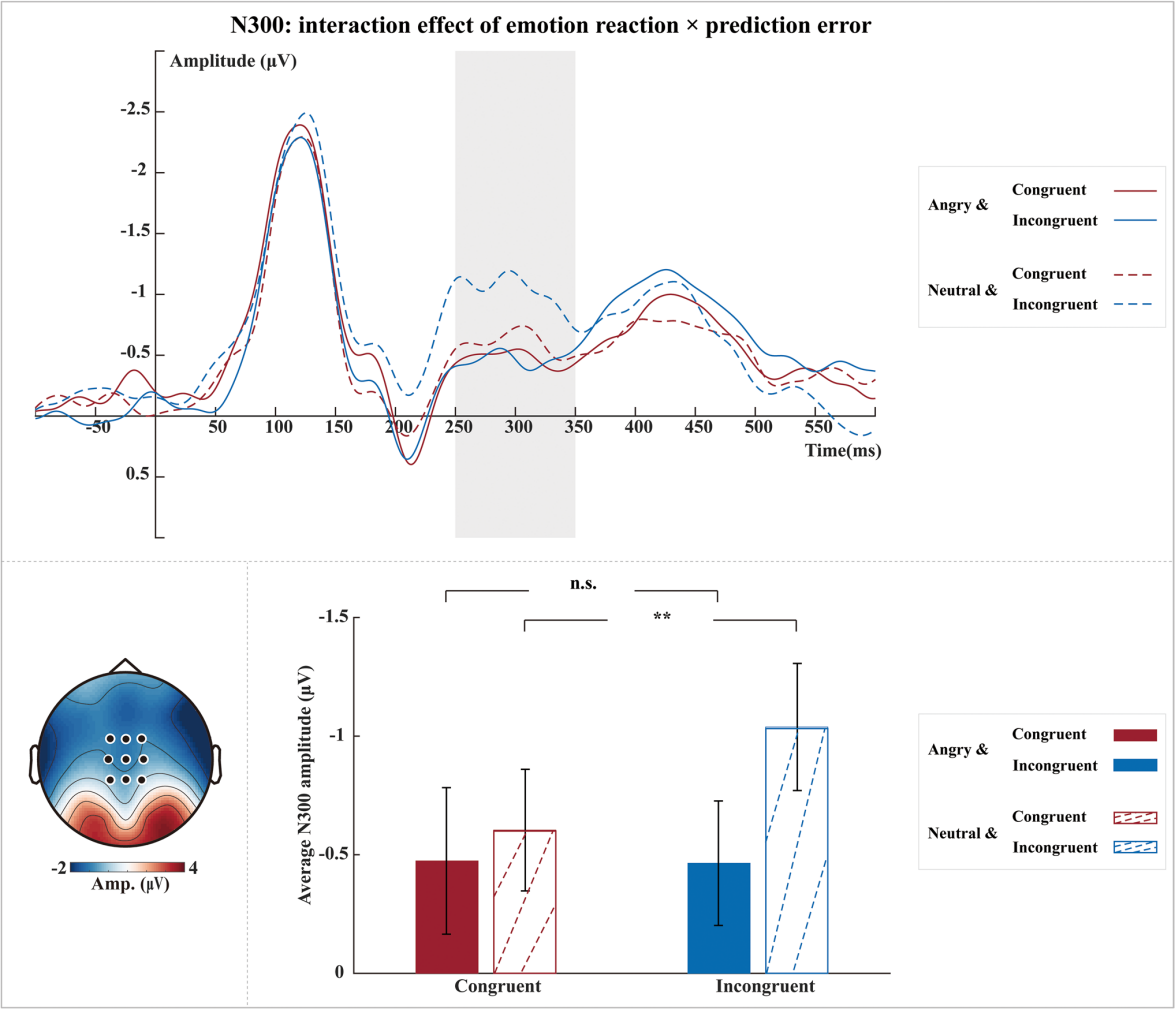
Interaction effect of emotion reaction × prediction error on N300. Grand-averaged ERP waveforms of N300 per condition (angry-congruent, neutral-congruent, angry-incongruent, and neutral-incongruent) (top). Waveforms were calculated by averaging the data at electrodes FCz, FC1, FC2, Cz, C1, C2, CPz, CP1, and CP2 (see black dots in scalp map). The shaded rectangle visualizes the time window from which the average ERP amplitude was extracted (250–350 ms). The topographic map was calculated by averaging the data of all conditions within a time window of 250–350 ms after the onset of the probe image (bottom left). Bar plots (bottom right) illustrate the mean and standard SE across participants of the average N300 amplitude per condition. **p< 0.01, n.s.: non-significant.

Further investigation of the observed interaction effect of emotion reaction × prediction error on N300 revealed a significant simple effect of prediction error for the neutral reaction (*t*(27) = 3.87,*p*= 0.001) as expected, but somewhat surprisingly not for the angry reaction (*t*(27) = -0.08,*p*= 1.000). More specifically, compared with neutral videos (-0.60 ± 1.38 µV), angry videos (-1.04 ± 1.44 µV) resulted in the subsequent neutral reaction eliciting larger N300 amplitudes (Fig. 4).

## 4 Discussion

The goals of the present study were to test first, whether observers of a social interaction derive predictions about its outcome and second, whether these predictions influence how information about the outcome is processed. Our study used a novel paradigm that measures the impact of viewing the initial stages of a social interaction on how the final stages are processed. We manipulated the prediction context in two different ways, by varying prediction strength and prediction error.

At the behavioral level, the accuracy of appropriateness judgments was highest in the high prediction condition, followed by the mid-prediction condition, and lowest in the low prediction condition. Thus, our behavioral results show that participants were able to successfully judge the appropriateness of the emotional reaction (the still probe image) when the preceding video provided clear social cues (high prediction condition). Performance gradually diminished to guessing behavior when the context provided fewer emotional cues (mid and low prediction conditions). These results confirm our hypothesis that observing social interactions may lead to predictions about the outcome. At the neural level, observing an angry reaction elicited significantly larger N170 amplitudes than observing a neutral reaction. This emotion effect was only found in the high prediction condition (where the context in the preceding video was intact and clear), not in the mid and low prediction conditions. Moreover, we found that the high prediction condition elicited larger N170 amplitudes than the mid and low prediction conditions. This prediction effect was found only in response to angry reactions. Additionally, observing social interactions can trigger prediction error effects. We found that incongruent conditions elicited larger N300 amplitudes than congruent conditions. This prediction error effect was found only in neutral reactions, not in angry reactions. These results confirm our hypothesis that social predictions may influence the perceptual and neural processing of social interactions.

### 4.1 Emotion effect on the early component N170 depends on prediction strength

Our first neural finding was that observing social interactions containing dyadic bodies evoked a clear N170 response. Previous studies have shown that the N170 is a marker of visual body processing (Borhani et al., 2015;de Gelder et al., 2004;Lu et al., 2023;Meeren et al., 2005;Stekelenburg & de Gelder, 2004). Here, we extend these previous findings by showing that the N170 is sensitive not only to a single body but also to body expressions in interactions involving two agents. Hence, our results are consistent with findings about the primacy of social interactions (Abassi & Papeo, 2020). Concerning the sensitivity of the N170, we further observed that the N170 is stronger for angry compared to neutral expressions. This is consistent with our recent finding (Lu et al., 2023) and, more importantly, extends previously observed emotional expression effects from single images and single-body expressions to social interaction situations.

Our main finding here is that the emotional expression effects during observation of interactions are only seen in the high prediction condition. In other words, neural discrimination between angry and neutral interaction images, as reflected by the N170, was not evident when the preceding social context videos did not allow clear and intact emotion predictions (mid and low prediction conditions). Moreover, the aforementioned emotion effect in the high prediction condition did not differ significantly across prediction error conditions (i.e., no significant three-way interaction) and we did not observe any interaction effects between emotion reaction/prediction strength and prediction error. This suggests that our N170 result reflects a more intense general evaluation of the ‘closure’ of the interaction for the higher stakes angry interaction, regardless of whether this closure confirms or disconfirms the observer’s predictions.

An alternative interpretation of our result might be that predictions were impacted by emotional context, such that high predictability elicited larger N170 amplitudes than lower predictions for videos of angry body interactions. Our results are supported by a previous study, which found that the N170 amplitude for normal, inverted faces was significantly larger than that for scrambled, inverted faces (Civile et al., 2018). This finding suggests that the N170 amplitude is more sensitive to normal stimuli compared to scrambled stimuli and that its sensitivity to stimulus information depends on the context. In summary, our results indicate that the N170 is not only responsive to social predictions triggered by the videos but also to the specific emotional content.

It is noteworthy that the N170 was preceded by a clear ERP peaking around 125 ms post stimulus onset. This early response may be attributed to sensory processing or early attentional mechanisms related to the image stimuli; however, we found no effect on it, suggesting that effects of emotion and prediction operate more reliably during later time windows starting with the N170.

### 4.2 Prediction error effect on the late component N300 depends on emotional whole-body interaction

Next, we found an effect of prediction error on the processing of observed social interactions, as reflected by the N300, in line with our expectations and previous results relating the N300 to higher-order visual prediction errors (Chen et al., 2022). More specifically, enhancements of the N300 have been related to unexpected and violating conditions compared to expected and confirming conditions (Baker et al., 2023;Kumar et al., 2021;Truman & Mudrik, 2018). In line with these studies, we found a prediction error response (incongruent > congruent) for social interactions. Interestingly, this effect was only significant when the emotional reaction was neutral, indicating that neutral reactions may violate emotion predictions more strongly than angry ones. Possibly, participants felt their predictions were more violated when the agent’s reaction showed a neutral body expression compared to an angry body expression. In sum, our N300 results indicate that the appropriateness of the reaction to an emotional interaction was extracted in the time window of the N300 (or 250–350 ms post-stimulus onset) in our study. Unexpectedly, we found no effect of prediction strength on prediction error responses in the N170 or N300, suggesting that these error responses do not necessarily depend on the availability of social predictions.

Our results underscore the notion that social interactions between conspecifics are rapidly processed in the presence of perceptual predictions, consistent with recent literature pointing in the same direction. The study of the perceptual basis of social interaction is a relatively new field of research. Traditionally, research on social interaction has mostly appealed to complex mental processes sustaining our ability to decipher intentions conveyed through facial expressions and bodily movements at stake in interactions. The pioneering research ofHeider and Simmel (1944)demonstrated that humans possess the ability to discern intricate details about others’ interactions based solely on basic visual cues. More recently, there has been a notable shift in both theory and empirical investigation toward recognizing the pivotal role of visual cues in understanding social behaviors (de Gelder & Poyo Solanas, 2021;McMahon & Isik, 2023). Yet the literature is still very limited and only in the last decade explicit arguments in favor of a novel orientation of neuroscience away from high-level cognition have been brought forward (for a review seePoyo Solanas & de Gelder, 2025). Given the obvious evolutionary importance of social interaction, its perception may rely on fast, automatic, and visually driven processes, rather than complex mental models.

## 5 Conclusion

Our results show that observing a social interaction generates perceptual predictions about how the behavior of the agents and these predictions affect cortical processing in the time window of the N170. The strength of this prediction effect measured at the final image is a function of how informative the preceding video is. This signifies that combined emotional expressions of interacting agents can be rapidly detected in early processing stages and that social interaction predictions influence information processing at perceptual and neural levels. Later prediction errors are reflected in the N300 amplitude, and this prediction error processing is most pronounced when observing a neutral bodily reaction. This suggests that later prediction may involve deeper cognitive processing reckoning with the emotional context in social interactions.

Our study offers new insights into prediction processes during social interaction, especially when it involves emotional information. Our experimental design, using videos and images of social interactions as stimuli, made the lab experiment more engaging and realistic. Our findings also prompt us to consider how social context and prior information influence our judgments in daily interactions, and how our evaluations are affected by the outcomes following predictions.

## Supplementary Material

Supplementary Material

## Data Availability

The data and code that support the findings of this study are available on request from the corresponding author (Beatrice de Gelder), pending approval from the researcher’s local ethics committee and a formal data-sharing agreement.
