# Supplementary material for: Behavioral and neural evidence for perceptual predictions in social interactions
Source: Imaging Neurosci (Camb). 2025 Jun 11;3:IMAG.a.38. doi: 10.1162/IMAG.a.38 (PMC12319869; doi:10.1162/IMAG.a.38)
Supplement: Supplementary Material [file imag.a.38_supp.pdf]

Main effect of prediction error on N170 and N400

Analysis for main effects revealed a significant effect of *prediction error* (i.e., pooled across *reaction emotion* and *prediction strength*) on N170 and N400. More specifically, the incongruent condition elicited smaller N170 amplitudes and larger N400 amplitudes than the congruent condition (N170: incongruent: -0.51 ± 1.76 μV, congruent: -0.78 ± 1.90 μV, F (1, 27) = 7.13, p = 0.013, η_p^2 = 0.21; N400: incongruent: -0.88 ± 1.36 μV, congruent: -0.71 ± 1.35 μV, F (1, 27) = 5.79, p = 0.023, η_p^2 = 0.18).

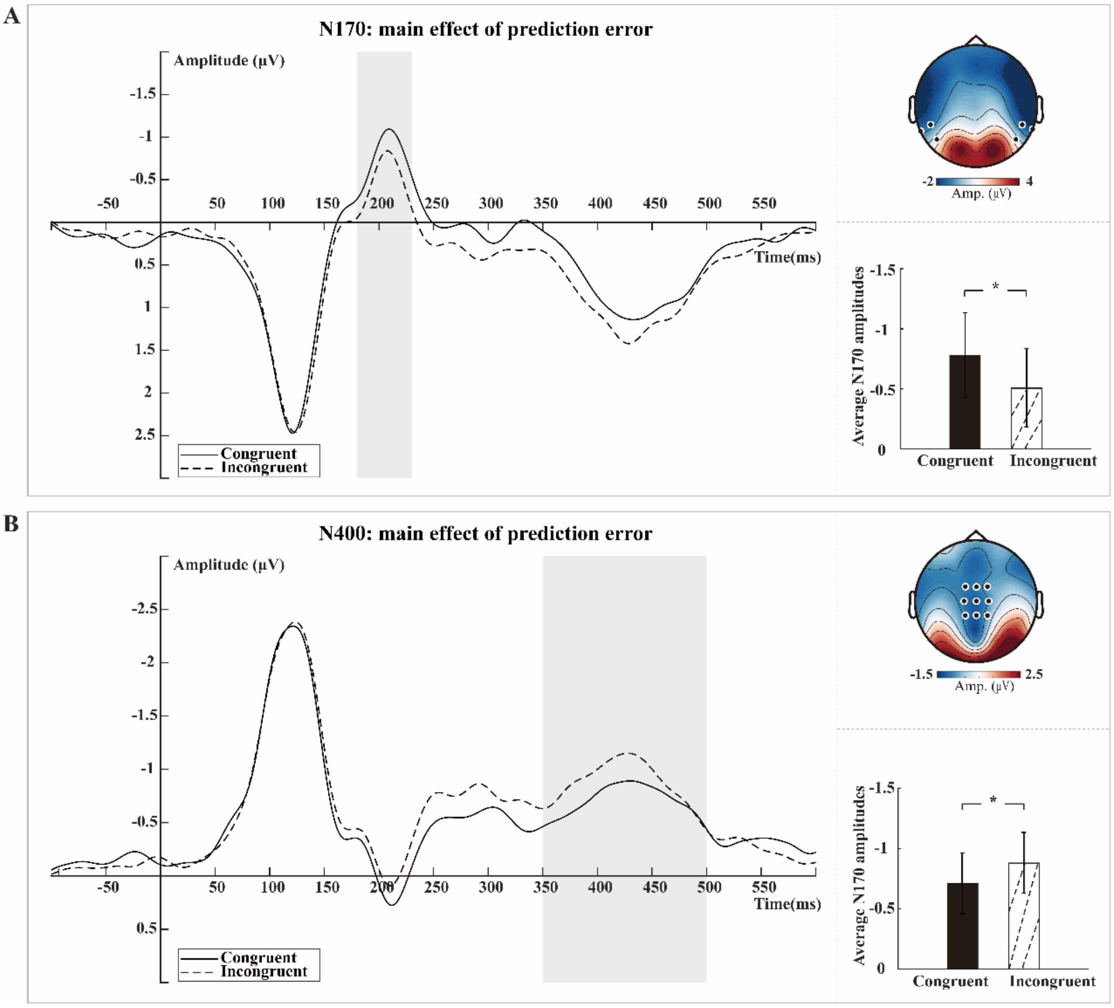

Fig. S1. A-B. Main effect of *prediction error* on N170 and N400. Grand averaged

ERPs are depicted per condition (congruent and incongruent) for N170 and N400 components separately (left). The shaded rectangle visualizes the time window (180-230ms for N170, and 350-450ms for N400) from which the average ERP amplitude was extracted. The highlighted black dots on the topographic map (right top) represent the electrodes from which the grand-averaged ERP for each component was extracted across all conditions. Bar plots (right bottom) illustrate the mean and SE across participants of each component's amplitude per condition. *: $p < 0.05$

Main effect of prediction strength on N300 and N400

We further observed a significant main effect of *prediction strength* (i.e., pooled across *reaction emotion* and *prediction error*) on N300 and N400. More specifically, high prediction resulted in subsequent still images eliciting smaller N300 amplitudes and N400 amplitudes, compared with mid and especially low prediction (N300: high: -0.26 ± 1.51 µV, mid: -0.62 ± 1.74 µV, low: -1.05 ± 1.33 µV, F (1, 27) = 9.54, p = 0.001, η_p^2 = 0.26; N400: high: -0.26 ± 1.51 µV, mid: -0.84 ± 1.53 µV, low: -1.12 ± 1.31 µV, F (1, 27) = 10.13, p = 0.001, η_p^2 = 0.27).

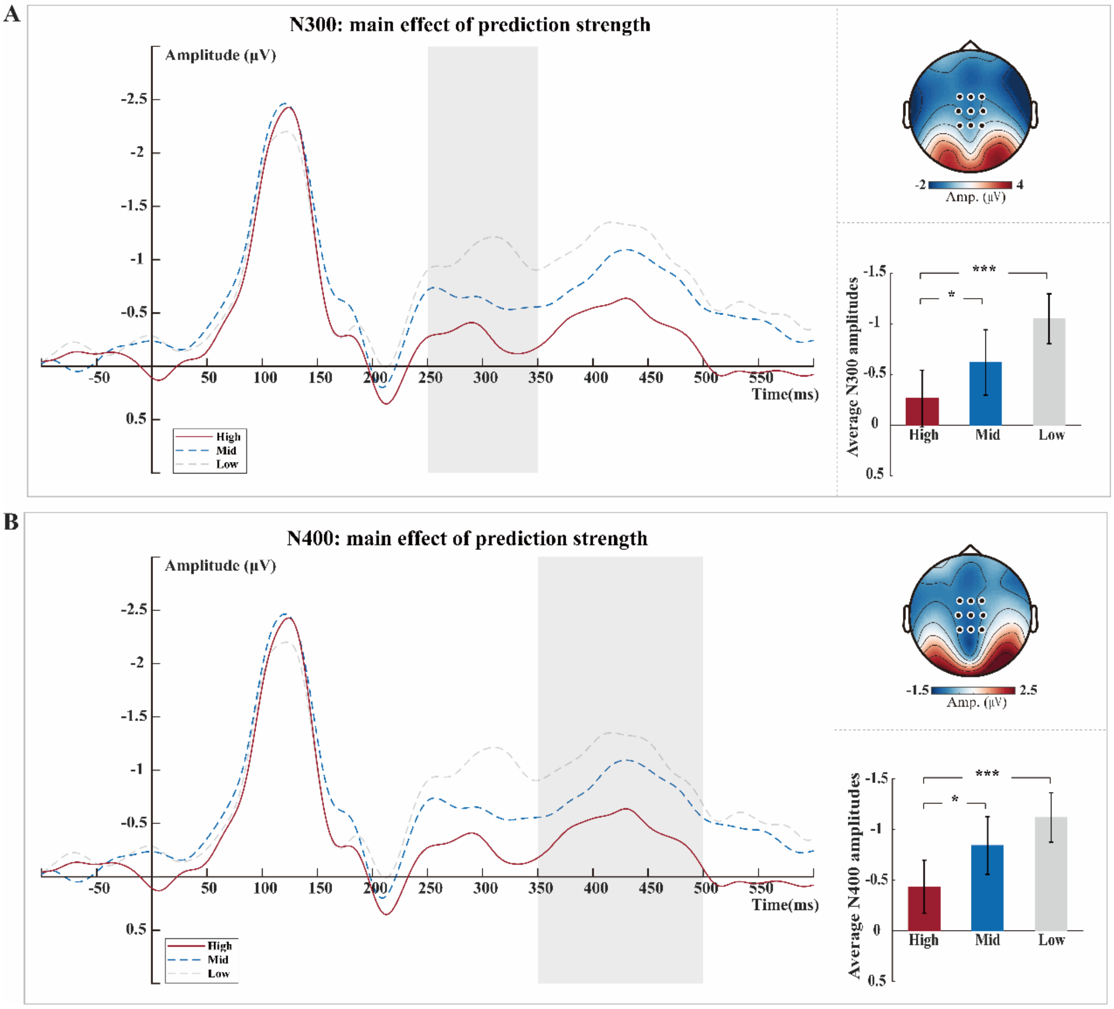

Fig. S2. A-B. Main effect of *prediction strength* on N300 and N400. Grand averaged ERPs are depicted per condition (high, mid and low) for N300 and N400 components separately (left). The shaded rectangle visualizes the time window (250-350ms for N300, and 350-450ms for N400) from which the average ERP amplitude was extracted. The highlighted black dots on the topographic map (right top) represent the electrodes from which the grand-averaged ERP for each component was extracted across all conditions. Bar plots (right bottom) illustrate the mean and SE across participants of each component's amplitude per condition. ***: $p < 0.001$, *: $p < 0.05$
